# Follistatin-like protein 1 promotes inflammatory reactions in nucleus pulposus cells by interacting with the MAPK and NFκB signaling pathways

**DOI:** 10.18632/oncotarget.17400

**Published:** 2017-04-24

**Authors:** Yi Liu, Jianlu Wei, Yunpeng Zhao, Yuanqiang Zhang, Yingguang Han, Bin Chen, Kaiyuan Cheng, Jialin Jia, Lin Nie, Lei Cheng

**Affiliations:** ^1^ Department of Orthopedics, Qilu Hospital of Shandong University, Jinan, China; ^2^ Department of Orthopedics, Affiliated Hospital of Shandong Academy of Medical Sciences, Jinan, China

**Keywords:** FSTL1, lumbar disc herniation, Inflammation, MAPK signaling, NFκB signaling

## Abstract

**Objective:**

Follistatin-like protein 1 (FSTL1) is a well-known mediator of inflammation. Intervertebral disc disease is an inflammatory disorder. Here, we investigated the role of FSTL1 in the intervertebral discs inflammation.

**Methods:**

Expression of FSTL1 in nucleus pulposus tissues from rats and human was determined by immunohistochemistry staining and western blot analysis. The expression levels of tumor necrosis factor-α (TNF-α), interleukin1-β (IL-1β) and matrix metalloproteinase 13 (MMP-13) in human and rat nucleus pulposus tissues were measured by immunohistochemistry staining. The mitogen-activated protein kinase (MAPK) and nuclear factor-kappa B (NFκB) signaling pathways were detected by western blotting.

**Results:**

FSTL1 serum levels were significantly increased in lumbar disc herniation patients and had a positive correlation with Visual Analogue Scores. Additionally, FSTL1 expression was significantly increased in extrusion group compared with protrusion and control groups. Furthermore, FSTL1 expression was significantly increased in intervertebral disc degeneration models of rats. Immunohistochemistry staining demonstrated that the levels of TNF-α, IL-1β and MMP-13 were increased in the pathogenesis of intervertebral disc disease. Recombinant human FSTL1 significantly increased the production of proinflammatory cytokines *in vitro*. In addition, FSTL1 promoted inflammation by activating c-Jun N-terminal kinase (JNK), extracellular regulated protein kinases 1/2(ERK1/2) and NFκB signaling.

**Conclusions:**

These data imply that FSTL1 expression was increased in the pathogenesis of intervertebral disc disease. Importantly, FSTL1 promoted inflammatory catabolism in the nucleus pulposus by activating JNK, ERK 1/2/MAPK and NFκB signaling.

## INTRODUCTION

Lumbar disc herniation (LDH) is caused by intervertebral disc degeneration, trauma, spinal structural abnormalities, and genetic factors, and is prevalent in specific ethnicities. The current treatment mainly focuses on relieving the pain instead of inhibiting the pathogenesis of intervertebral disc degeneration. Normal discs balance the anabolism and catabolism of the extracellular matrix to maintain tissue homeostasis. When intervertebral disc degeneration occurs, inflammatory cytokines, which regulate matrix metabolism, were significantly increased. LDH is characterized by the destruction and/or disintegration of the annulus fibrosus that leads to piercing of the central nucleus pulposus increased expression of inflammatory cytokines which may directly cause severe back pain or sciatica without nerve root compression [1].

Follistatin-like protein 1 (FSTL1), also known as transforming growth factor-1β stimulated clone 36 (TSC-36) or follistatin-related protein (FRP), is a soluble glycoprotein that was first cloned from the mouse osteoblastic MC3T3-E1 cell line [2]. FSTL1 is an extracellular matrix protein which is widely expressed in all eukaryotic cells except for peripheral lymphocytes [3, 4]. FSTL1 participates in regulation of cell proliferation, apoptosis, metabolism, cell differentiation, the immune response and endocrine function [5–7]. FSTL1 is also associated with inflammation [8, 9]. It is widely known that mitogen-activated protein kinase (MAPK) and nuclear factor-kappa B (NFκB) signaling play decisive role in lumbar disc inflammation. It is known that FSTL1 increases the expression of inflammatory mediator expression by activating the NFκB signaling in HEK293 cell line [10] and by activating of NFκB and c-Jun N-terminal kinase (JNK) signaling in both adipocytes and macrophages [11]. However, the role of FSTL1 in the pathogenesis of LDH is still not clear. Here, we determined FSTL1 expression level in the pathogenesis of LDH using human disc samples, human primary nucleus pulposus cells, and a rat needle punch model.

## RESULTS

### FSTL1 and related proinflammatory cytokines are highly expressed in the serum and disc tissue from LDH patients

**Figure 1:**
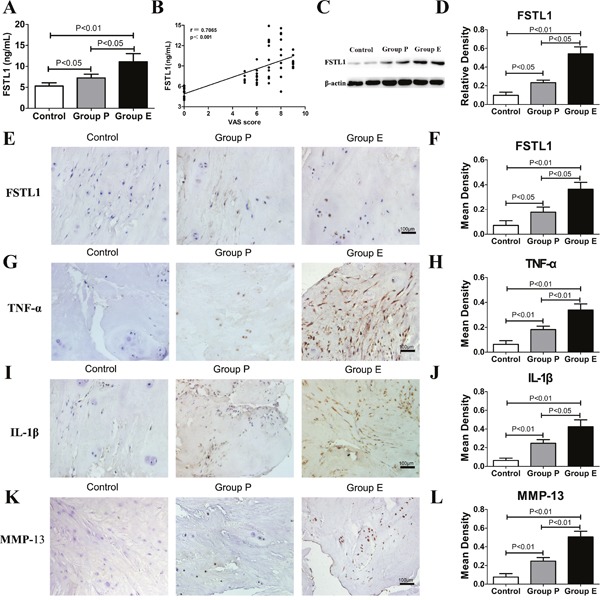
FSTL1 and relative inflammatory cytokines levels were increased in LDH patients **(A)** The serum level of FSTL1 in LDH patients was higher than that of the scoliosis controls. Sixty-four peripheral blood samples (Group P, n = 25; Group E, n=25; control group, n=14) were obtained for ELISA. **(B)** The Pearson correlation coefficients were positive for the correlation of VAS pain scores and FSTL1 levels in human serum (r=0.7065, P<0.001). **(C-D)** Protein expression of FSTL1 in the intervertebral disc tissue of LDH patients was higher than that of the scoliosis controls, as measured and analyzed by western blot assay (Group P, n = 25; Group E, n=25; control group, n=7). **(E-L)** Expression of FSTL1, TNF-α, IL-1β and MMP-13 in human degenerated intervertebral disc tissues (group E and group P) were higher than that of the controls. Fifty-seven disc tissues samples (Group P, n = 25; Group E, n=25; control group, n=7) were obtained for immunohistochemistry and mean density analysis. Magnification, 200×; scale bars, 100μm. Each section was examined independently by two investigators in a blinded manner.

Serum FSTL1 levels are notably increased in rheumatoid arthritis and osteoarthritis patients [12, 13]. In this study, enzyme-linked immune sorbent assay (ELISA) and western blots were used to investigate FSTL1 expression in LDH patient samples and scoliosis patient samples (controls). ELISA results showed that the serum FSTL1 levels in the protrusion group (7.240±0.905 ng/mL) and the extrusion group (11.080±1.961 ng/mL) were significantly higher than that of the scoliosis controls (5.301±0.779 ng/mL) (Figure 1A). Pearson correlation coefficients showed that the visual analogue scores (VAS) was positively correlated with FSTL1 levels (r=0.7065, p<0.001; Figure 1B). As shown in Figure 1C and 1D, the relative expressions of FSTL1 protein in group P and group E were higher than that in the control group.

To investigate the expression of FSTL1, tumor necrosis factor-α (TNF-α), interleukin1-β (IL-1β) and matrix metalloproteinase-13 (MMP-13), the disc tissue of LDH patients and scoliosis patients were stained using immunohistochemical staining. As shown in Figure 1E and 1F, a number of nucleus pulposus cells expressing FSTL1 were observed in Group P and Group E. The numbers of FSTL1-positive cells in Group E were significantly higher than that of other groups. TNF-α and IL-1β exacerbate LDH inflammatory processes, and are believed to be key players during disc degeneration. MMP-13 is mainly involved in the degradation of collagen II and accelerates the degeneration of nucleus pulposus tissue. In samples from Group E and Group P study participants, the inflammatory regions showed many nucleus pulposus cells and inflammatory cells secreting TNF-α, IL-1β and MMP-13 (Figure 1G, 1I and 1K). In the control group, disc tissue had clear structures, intact tissue morphology, intact nuclei, normal cell morphology, no tears in the annulus fibrosus tissue, and lower secretion of TNF-α, IL-1β and MMP-13 than that of other groups. The positive expression area and expression intensity of TNF-α, IL-1β and MMP-13 in group E were significantly higher than that in the other two groups (Figure 1H, 1J and 1L).

### FSTL1 and related proinflammatory cytokines are highly expressed in intervertebral disc degeneration models of rats

**Figure 2:**
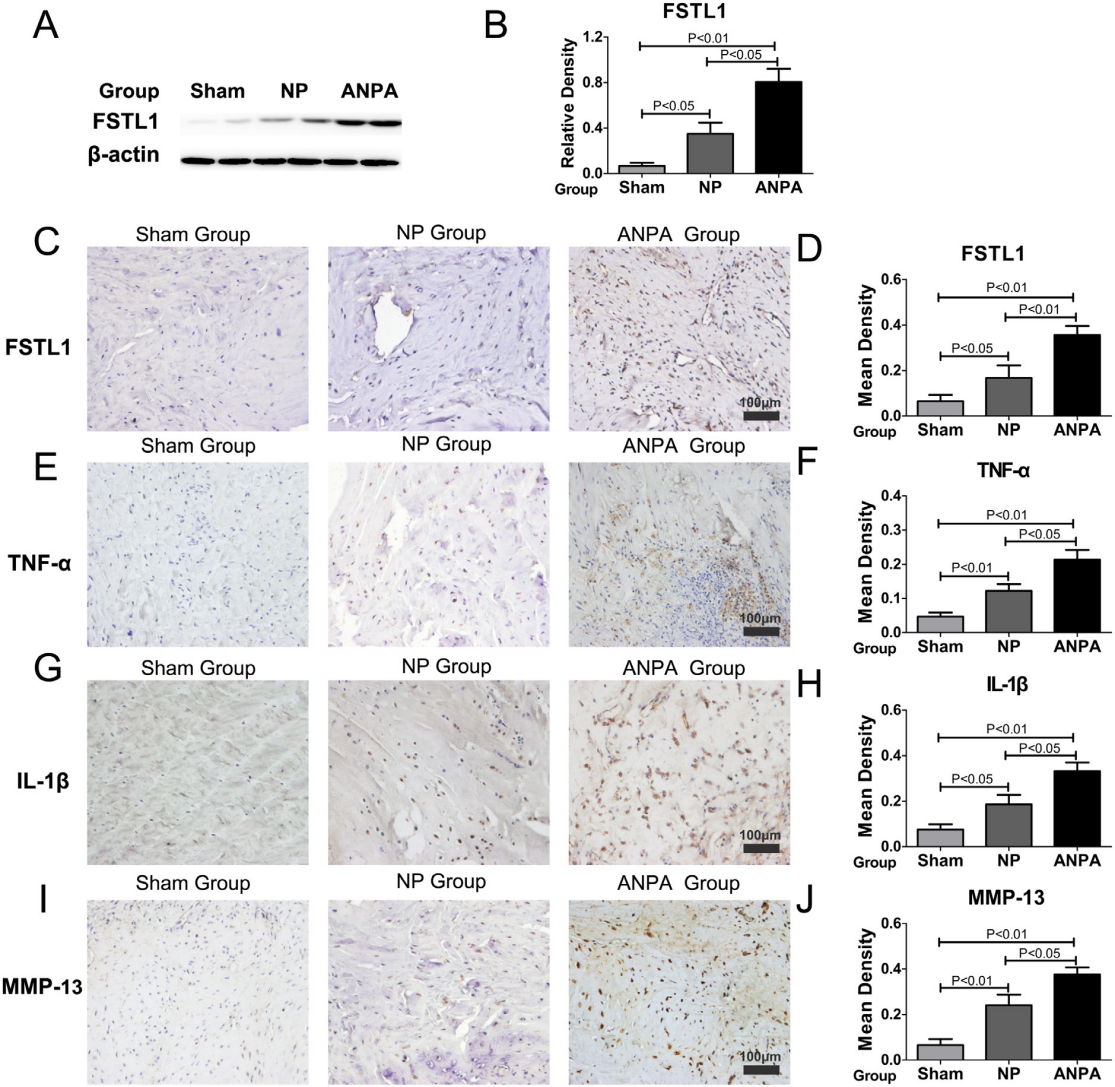
FSTL1 is highly expressed in the rat intervertebral disc degeneration model **(A)** The relative expression of FSTL1 in NP and ANPA groups were significantly increased compared with that in the sham group, as assayed by western blotting. **(B)** The expression level of FSTL1 in the ANPA group was significantly increased versus that of the NP and sham groups **(C-D)** The expression and mean density of FSTL1 in the ANPA and NP groups were increased more so in the ANPA group than the NP group as tested by immunohistochemistry staining analysis. **(E-J)** The expression levels of TNF-α, IL-1β, MMP-13 in the NP and ANPA groups were dramatically increased and the mean densities of these inflammatory cytokines were significantly elevated in the ANPA group compared with the other groups as detected and quantified by immunohistochemistry staining analysis. Twenty-four male Wistar rats disc tissues samples (ANPA group, n = 8; NP group, n=8; Sham group, n=8) were obtained for western blot and immunohistochemistry analysis. Magnification, 200×; scale bars, 100μm. Each section was examined independently, in a blinded manner, by two investigators.

Compared with the sham group, the relative expressions of FSTL1 in the NP and ANPA groups were significantly increased (Figure 2A and 2B), detected by western blotting. A series of cascade reactions occurred in the disc tissue samples from the ANPA group: the tissues had lost their normal form, nucleus pulposus cells secreted a large number of inflammatory mediators that recruited surrounding inflammatory cells, a large number of inflammatory cells infiltrated the nucleus pulposus tissue, and the inflammatory response was activated. More FSTL1-producing nucleus pulposus cells were observed in the intervertebral disc degeneration groups (Figure 2C). The mean density of FSTL1 was increased in the ANPA group compared to that in the NP group (Figure 2D). The expression of TNF-α, IL-1β and MMP-13 in inflammatory regions was dramatically increased in the NP and ANPA groups (Figure 2E, 2G and 2I). Quantitative analysis indicated that the mean density of TNF-α, IL-1β and MMP-13 was significantly elevated in the ANPA group compared to other groups (Figure 2F, 2H and 2J). In accordance with the patients in Group E, the rats in the ANPA group exhibited increased expression of TNF-α, IL-1β and MMP-13 compared with the NP group (Figure 2F, 2H and 2J).

### FSTL1 expression was induced by recombinant human TNF-α (rhTNF-α) and inflammatory cytokine expression was up-regulated by recombinant human FSTL1 (rhFSTL1) in human nucleus pulposus cells

**Figure 3:**
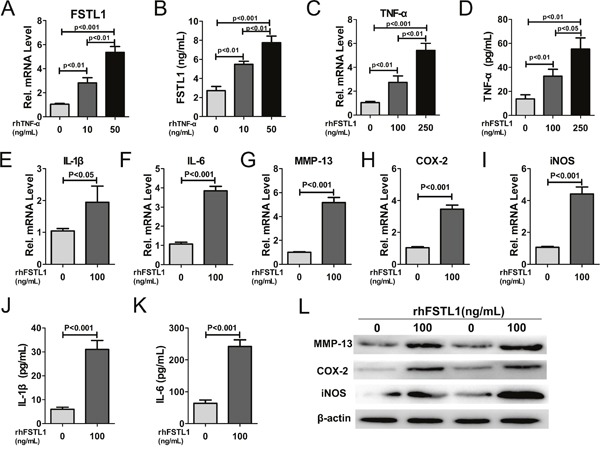
FSTL1 expression was stimulated by TNF-α and FSTL1 up-regulated the expression of TNF-α, IL-1β, IL-6, MMP-13, COX-2, and iNOS in human nucleus pulposus cells **(A)** rhTNF-α significantly enhanced FSTL1 mRNA expression in a concentration-dependent manner compared with untreated human nucleus pulposus cells. Nucleus pulposus cells were cultured and stimulated with 0, 10, and 50 ng/mL of rhTNF-α for 24 h and FSTL1 expression was detected by real-time PCR. **(B)** FSTL1 levels in the cell culture supernatant significantly increased in a dose-depended manner with TNF-α treatment, as tested by ELISA assay. The cell supernatant was collected after stimulation of with 0, 10, and 50 ng/mL of rhTNF-α for 48 h. **(C-D)** The expression level of TNF-α was significantly increased under the stimulation of rhFSTL1 in a concentration-dependent manner. Nucleus pulposus cells were collected and analyzed by real-time PCR after being stimulated with 0, 100, and 250 ng/mL of rhFSTL1 for 24 h. Cell supernatant was collected and used in an ELISA assay after a 48 h stimulation. **(E-I)** The mRNA expression levels of IL-1β, IL-6, MMP-13, COX-2, and iNOS were significantly increased in human nucleus pulposus cells stimulated by rhFSTL1 compared with the control group, as measured by real-time PCR. RNA was extracted from nucleus pulposus cells cultured with 0 and 100 ng/mL of rhFSTL1 for 24 h. **(J-K)** IL-1β and IL-6 levels were significantly increased in rhFSTL1-stimulated nucleus pulposus cells after 48 h, as assayed by ELISA. **(L)** Expression levels of MMP-13, COX-2, and iNOS in nucleus pulposus cells were detected by western blot. Total protein was extracted from nucleus pulposus cells stimulated with 0 and 100 ng/mL rhFSTL1 for 48 h. Each experiment was repeated in triplicate and the values are given as the mean ± SD.

FSTL1 is induced by various inflammatory cytokines [4, 8]. To determine whether TNF-α could induce FSTL1 expression in human nucleus pulposus cells, the cells were stimulated with rhTNF-α. As shown in Figure 3A and 3B, TNF-α significantly increased FSTL1 secretion compared to untreated cells. Next, we analyzed the ability of FSTL1 to regulate the expression of inflammatory cytokines in nucleus pulposus cells. Quantitative analysis showed that rhFSTL1 significantly promoted mRNA overexpression of TNF-α, IL-1β, interleukin6 (IL-6), cyclooxygenase-2 (COX-2), MMP-13, and inducible nitric oxide synthase (iNOS) in nucleus pulposus cells (Figure 3C–3I). IL-1β and IL-6 protein levels in the supernatant were analyzed by ELISA and COX-2, MMP-13, and iNOS protein levels in nucleus pulposus cells were detected by western blotting. Consistent with their mRNA expression levels, the protein expression of corresponding inflammatory cytokines was markedly up-regulated by rhFSTL1 compared to the untreated group (Figure 3J–3L).

### FSTL1 promoted the expression inflammatory cytokines by activating the JNK, extracellular signal-regulated protein kinase1/2 (ERK1/2) and NFκB signal pathways *in vitro*

**Figure 4:**
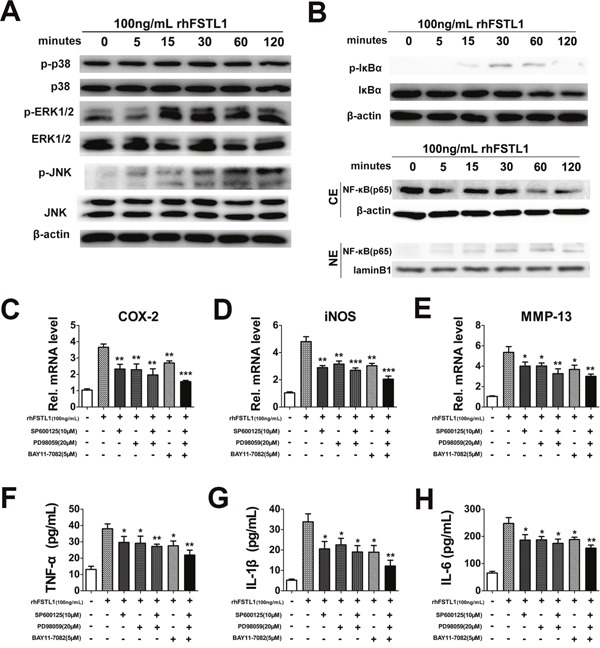
FSTL1 activated the JNK/MAPK, ERK1/2/MAPK and NF-κB signaling pathways; blocking the JNK/MAPK, ERK1/2/MAPK and NFκB pathways inhibited FSTL1-mediated expression of cytokines **(A)** rhFSTL1-induced phosphorylation of JNK and ERK1/2 were remarkably increased after 15 min of stimulation and maintained to 120 min, while the expression of p-p38 and p38 did not change obviously, as measured by western blot. Protein was collected from human nucleus pulposus cells stimulated with 100 ng/mL rhFSTL1 at 0, 5, 15, 30, 60, and 120 min time points. **(B)** rhFSTL1-induced phosphorylation of IκBα in nucleus pulposus cells was increased in nucleus pulposus cells with IκBα degradation. rhFSTL1-induced expression of NF-κB (p65) was increased in the nucleus (NE) with degradation in the cytoplasm (CE). Nucleus pulposus cells were stimulated by rhFSTL1 as in (B) and protein was collected to perform a western blot assay. **(C-E)** rhFSTL1-induced up-regulated expression of COX-2, iNOS, MMP-13 was remarkably diminished by inhibition of the JNK, ERK1/2/MAPK and/or NF-κB signaling pathways, as detected by real-time PCR. Nucleus pulposus cells were cultured with 100 ng/mL rhFSTL1 in the presence or absence of 10 μM SP600125, 20 μM PD98059 and 5 μM BAY11-7082 for 24 h. A combined application of all three inhibitors showed an enhanced blocking effect compared with the rhFSTL1-treated group. **(F-H)** The up-regulated levels of TNF-α, IL-1β and IL-6, stimulated by rhFSTL1, were reduced by inhibitors of the JNK, ERK1/2/MAPK and/or NF-κB signaling pathways. After 48 h the cell supernatant was collected from nucleus pulposus cells and analyzed using an ELISA. *P < 0.05, **P < 0.01. Each experiment was repeated in triplicate and the values are given as the mean ± SD.

It is known that both the MAPK and NFκB signaling pathways have a critical role in intervertebral disc degeneration [14, 15]. To investigate whether FSTL1 affected these signaling pathways in human nucleus pulposus cells, we treated cells with rhFSTL1 for different periods of time and then measured protein expression using western blot assays. The expression levels of p-JNK and p-ERK1/2 in nucleus pulposus cells increased 15 min after rhFSTL1 treatment, peaked at the 30 min time point and remained at this level till the 120 min time point (Figure 4A). Moreover, as Figure 4A shows, there were no obvious differences between the expressions of p-p38 in rhFSTL1-treated nucleus pulposus cells, which suggested that rhFSTL1 did not activate p38 pathways. The expression level of phosphorylated I-Kappa-B-Alpha (IκBα) in nucleus pulposus cells increased 15 min after rhFSTL1 treatment, peaked at the 30 min time point and then slowly decreased after 60 min (Figure 4B). While IkBα had a slow decreasing trend in cells treated with rhFSTL1 for 5 min. We also observed that the expression of P65 in the nuclei of nucleus pulposus cells dramatically increased 5 min after rhFSTL1 treatment and peaked at 30 min. However, the level of cytoplasmic P65 decreased after rhFSTL1 treatment. Collectively, these data show that rhFSTL1 activated the JNK, ERK1/2 and NFκB signal pathways in nucleus pulposus cells.

To further verify whether rhFSTL1 played a pro-inflammatory role through the JNK, ERK1/2 and NFκB signaling pathways, nucleus pulposus cells were treated with 100 ng/mL rhFSTL1 with or without of pharmacological JNK, ERK1/2 and NFκB inhibitors for RT-PCR and ELISA assays. The JNK, ERK1/2 and NFκB pathways were specifically blocked by SP600125, PD98059 and BAY11-7082, respectively. As shown in Figure 4C-4E, inhibition of the JNK, ERK1/2 and/or NFκB pathways significantly suppressed the rhFSTL1-induced expression levels of COX-2, iNOS and MMP-13. The rhFSTL1-induced expressions of TNF-α, IL-1β and IL-6, as detected by the ELISA assay, were dramatically reduced by blocking the MAPK and/or NFκB pathways (Figure 4F-4H). These experiments suggest that the JNK/MAPK, ERK1/2/MAPK and NFκB pathways mediated the pro-inflammatory action of rhFSTL1 in human nucleus pulposus cells.

## DISCUSSION

Lumbar disc degeneration lead to a series of changes in tissue physiology, biochemistry, morphology and function, including excessive breakdown of the extracellular matrix by proteases, loss of collagens and proteoglycans, degenerative fibrillation, decreased water content and increased cell senescence and death [16, 17]. These changes reduced the load-bearing ability of the disc and result in decreased osmotic pressure and impact cell mechanobiology. The invertebral disc is the largest avascular tissue and is supplied with nutrients mainly by infiltration of the surrounding tissues. According to auto-immune theory, the pathological process of intervertebral disc degeneration is a type of autoimmune reaction [18]. The nucleus pulposus isolated from the host immune system exists in an immuno-privileged state. When disc herniation occurs, the nucleus pulposus makes contact with the surrounding tissue fluid, causes immune dysfunction and increases the secretion of pro-inflammatory cytokines. The recruitment and accumulation of inflammatory cells and secretion of inflammation mediators leads to low back pain or sciatica. FSTL1 is related to the pathogenesis of human inflammatory and autoimmune diseases, including osteoarthritis, rheumatoid arthritis, reactive arthritis, ulcerative colitis, systemic lupus erythematosus [4, 12, 19]. The level of anti-FRP antibodies is correlated with inflammatory signs such as increasing erythrocyte sedimentation rate (ESR) and over-expression of serum C-reactive protein (CRP) in rheumatoid arthritis patients [19]. FSTL1 expression in the lumbar disc has not been reported and whether FSTL1 plays a pro-inflammatory role in the pathological processes of lumbar disc herniation is unknown. Serum FSTL1 levels were measured for the three groups of study participants and we found that FSTL1 secretion was increased in group E compared with the control group. The VAS score system is commonly used clinically to describe the patient's subjective feeling of pain. A pronounced, positive correlation was observed between serum VAS and serum FSTL1 levels which suggests that FSTL1 may be a new marker for LDH diagnoses.

To verify the hypothesis further, we measured FSTL1 protein expression in tissues from the LDH patients and rats from the animal model using IHC staining and western blot. In both group E of LDH patients and the ANPA rat group, nucleus pulposus cells secreted large amounts of FSTL1 and nucleus pulposus tissue also recruited a large number of inflammatory cells which secreted large amounts of TNF-α and IL-1β. Western blot results showed that there was low expression of FSTL1 in the control group. Along with the increase of the degree of disc degeneration, FSTL1 protein expression in nucleus pulposus tissues increased (according to ELISA and immunohistochemistry results). Degenerative discs over-express many inflammatory cytokines [20], including TNF-α, IL-1β, COX-2, thus suggesting that inflammation participates in the degenerative cascade, causing the biochemical and biomechanical changes closely involved in degenerative intervertebral discs. A herniated disc recruits a large number of inflammatory cells, which is prodominant in the inflammatory process and causes the increase expression of TNF-α, IL-1β and other inflammatory mediators at the herniation site [21, 22]. Disc cells respond to TNF-α and IL-1β stimulation by reducing matrix protein synthesis and over-expressing matrix-degrading enzymes, resulting in catabolism [15, 20, 23]. This is a positive feedback loop process, after multiple cycles, disc degeneration continued to increase [20]. We also found that the content of inflammatory cytokines (TNF-α and IL-1β) increased significantly in group E and group P of patients and in the rat ANPA group and the rat NP group. We also tested whether MMP-13 was highly expressed in human degenerative intervertebral disc tissue and in the rat ANPA and NP groups. MMP-13 belongs to the matrix metalloproteinase family and is highly overexpressed in pathological situations such as human carcinomas, osteoarthritis and rheumatoid arthritis. In humans, the MMP-13 gene encodes collagenase 3 which is associated with the degradation of collagen [24]. These results show that FSTL1 is closely related to the intervertebral disc inflammation.

FSTL1 is composed of 308 amino acids and an N-terminal signal peptide which contains 20 amino acids. FSTL1 has a highly conserved protein structure across mammalian species and FSTL1 proteins share 91% of the common amino acid sequence of mice and humans and has two glycosylated isoforms with similar functional activity [25]. FSTL1 contains two domains: one is a follistatin-like domain which suggests that FSTL1 is classed as the secreted protein-acidic and rich in cysteine (SPARC) family of proteins; the other one is an non-functional extracellular calcium-binding domain [26]. FSTL1 is widely expressed in various tissues and organs and is secreted by endothelial cells, neurons, and the mesenchymal lineage of cells which include cardiomyocytes, osteocytes, adipocytes, chondrocytes, and fibroblasts [4, 19, 26, 27]. Different from other inflammatory cytokines, FSTL1 is not secreted by the hematopoietic lineage cells, such as monocytes/macrophages, T cells and B cells. Studies confirmed LPS, IL-1β, TNF-α, and IL-6 could induce FSTL1 expression increased in osteoblasts, adipocytes, chondrocytes, and human fibroblast-like synoviocytes [4, 11, 26]. In this study, the expression of the pro-inflammatory mediator, FSTL1, was up-regulated by rhTNF-α in a dose-dependent manner in nucleus pulposus cells. This suggests that FSTL1 participates in the intervertebral disc inflammatory process *in vitro*. Human nucleus pulposus cells treated with rhFSTL1 0, 100 and 250 ng/mL were assessed using western blot and PCR assays to detect the expression of pro-inflammatory cytokines (TNF-α, iNOS, IL-1β and COX-2) and matrix metalloproteinases (MMP-13). We observed that rhFSTL1 treatment significantly increased the expression levels of TNF-α, IL-1β, COX-2, MMP-13 and iNOS in nucleus pulposus cells. These results are show that FSTL1 expression in nucleus pulposus cells promotes inflammation by increasing the expression of inflammatory factors and matrix proteases.

**Figure 5:**
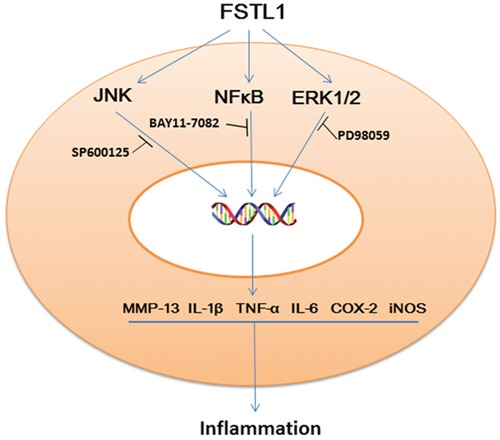
Schematic model of the proposed signal pathways of inflammation induced by FSTL1

Both NFκB and MAPK signaling are canonical pathways that participate in degeneration of the lumbar disc. FSTL1 activates NFκB signaling in HEK293 cell line [11], and functions as an important pro-inflammatory factor in the pathogenesis of osteoarthritis by activating the canonical NFκB pathway and enhancing synoviocyte proliferation [28]. In recombinant FSTL1-treated 3T3-L1 adipocytes and RAW264.7 macrophages, FSTL1 is a direct activator of JNK signaling [11]. In the present study, we also found that FSTL1 significantly up-regulated the phosphorylation of JNK/MAPK, ERK1/2/ MAPK but not p38/MAPK in FSTL1-stimulated nucleus pulposus cells. When we used JNK, ERK1/2and NFκB inhibitors to block the JNK, ERK1/2 and NFκB pathways, PCR and ELISA results showed that the expression of TNF-α, IL-1β, COX-2, and iNOS was decreased significantly when compared with rhFSTL1 treated group (Figure 5), suggesting that FSTL1 promotes the disc inflammation by increasing the activation of JNK/MAPK, ERK1/2/MAPK and NFκB pathways.

## MATERIALS AND METHODS

### Ethics statement

Fifty patients with single level lumbar disc herniation were enrolled between May 2016 and August 2016 at Qilu Hospital of Shandong University. Collection of lumbar disc tissue and peripheral blood samples of human was approved by the medical ethics regulations of the Medical Ethical Committee of Qilu Hospital of Shandong University. This study had been approved by the Qilu Hospital of Shandong University Review Board and a written informed consent document was requested and received from all study participants.

Animal experiments were approved by the Laboratory Animal Center of Shandong University and performed under the guidance and supervision of the International Guiding Principles for Animal Research.

### LDH patients and controls

All patients enrolled in this study were diagnosed according to the diagnostic criteria of LDH using surgical and imaging findings and were divided as follows: Group P (n = 25; 16 men and 9 women, 16 M/9F) in which imaging data showed intact annulus fibrosus; Group E (n = 25; 14 M/11F) in which imaging data showed ruptured annulus fibrosus. The inclusion criteria and the exclusion criteria were performed according to a previously described method [29, 30]. We also collected peripheral blood (PB) samples from the 50 LDH patients and 14 healthy controls (7 M/7F) before surgeries. Additionally, 7 scoliosis samples (2 M/5F) were used as controls for immunohistochemistry (IHC) staining and western blots. The characteristics of the research population are shown in Table 1.

**Table 1:** Baseline characteristics of study subjects

		Group P	Group E	Control	Control of Peripheral Blood
**Number**		25	25	7	14
**Age (year)**	**Mean**	38.31±8.24	41.35±7.81	16.75±4.56	26.5±4.07
	**Range**	26-50	29-56	13-26	20-34
**Male/Female**		16/9	14/11	2/5	7/7
**Average degeneration grading**	**GradeI**	III	IV	I	I

### Rats

Twenty-four male Wistar rats (8-week-old, 220–230 g) were obtained from the Laboratory Animal Center of Shandong University and were housed in a constant temperature environment with free access to food and water. Animals were randomly divided as follows: (1) Sham group (n = 8); (2) needle puncture group (NP group, n = 8) which was treated with needle puncture surgery; (2) needle puncture plus autologous nucleus pulposus application group (ANPA group, n = 8) which was treated with needle puncture plus autologous nucleus pulposus application surgery. The rats were used to establish the intervertebral disc degeneration model as previously described [31].

### Needle puncture model

Pentobarbital sodium (20 mg/kg; Sangon Biotech (Shanghai) Co., Ltd., Shanghai, China) was used to perform animal anesthesia. The skin surface of rats was cleaned and disinfected by 75% alcohol and the surgery was performed under the surgery microscope. After exposing the tail disc, a 20-gauge needle (Shinva Ande Healthcare Apparatus Co., Ltd, Shandong, China) was inserted into the middle of the disc (Co6-7) to a depth of 5 mm in a direction parallel to the direction of the end plate. Then, the needle was rotated 360°for 5 s, and the tail skin was closed layer by layer. In the sham group, after exposing the tail disc (Co6-7), the tail skin was closed without needle puncture surgery. The animals after the surgery were placed in a warm box and waited for their waking up.

### Needle puncture and autologous nucleus pulposus application model

After exposing the L5–6 facet joint and L5 nerve root, the autologous nucleus pulposus obtained from Co6-7, was placed on the exposed L5 nerve roots without compression at 4 weeks after needle puncture surgery. After the incision was closed, the animals were returned to their cages and carefully observed for 24 h. One week after surgery, all the rats were sacrificed by cervical dislocation; the discs were collected for future research.

### Immunohistochemistry

After being fixed with 4% paraformaldehyde, the nucleus pulposus tissues of both humans and rats were cut into 5-μm-thick slices for slide preparation. Tissues were incubated with theprimary antibodies as follows: goat anti-FSTL1 (1:200; Abcam plc, Cambridge, UK), rabbit anti- TNF-α, (1:300; Abcam), rabbit anti- MMP-13 (1:400; Abcam), rabbit anti- IL-1β, (1:200; Abcam). The slides after immunohistochemical staining were observed and analyzed by an electron microscope (Olympus, Tokyo, Japan). The results were quantified using the Image-Pro Plus 6.0 software (Media Cybernetics, Inc., USA). Each section was examined independently by two investigators in a blinded manner.

### Isolation and culture of human nucleus pulposus cells

Disc samples from patients were digested with trypsin (Gibco, Thermo Fisher Scientific Inc., NY, USA) and Type II collagen enzyme (Sigma-Aldrich, St. Louis, MO, USA). Dulbecco's Modified Eagle Medium: Nutrient Mixture F-12 (DMEM/F-12; Gibco, USA) culture media supplemented with 10% fetal bovine serum (FBS; Gibco, USA) was used to culture cells under standard incubation conditions (37°C, 5% CO2, 95% air). To determinate how the expression level of FSTL1 was affected by TNF-α, cells were exposed to 0, 10, and 50 ng/mL rhTNF-α(PeproTech, NJ, USA) for 24 h or 48 h. To observe the effect of FSTL1 of nucleus pulposus cells, the cells were pretreated with 0, 100, 250 ng/mL recombinant human FSTL1 (rhFSTL1, R&D systems Inc., MN, USA) for 24 h or 48 h. After differential treatment, the nucleus pulposus cells and supernatant samples were harvested for further analyses.

### Enzyme-linked immunosorbent assay

Serum was taken from peripheral blood that was obtained from 50 disc herniation patients and 14 healthy subjects and centrifugated at 3000 rpm/min for 10 minutes. The human serum and cell culture supernatant samples were collected and stored separately at -80°C. The FSTL1 serum level was measured by human FSTL1 ELISA kits (Bluegene, Shanghai, China). The serum levels of human TNF-α, IL-1 and IL-6 in cell culture supernatant were separately detected using human TNF-α, IL-1, IL-6 ELISA kits (Abcam, USA) according to the manufacturer's instructions.

### Western blot analysis

Nucleus pulposus cells and disc tissues lysates were prepared using RIPA buffer (Beyotime Biotechnology, Shanghai, China). Nuclear proteins were extracted using a nuclear protein extraction kit (BestBio, Shanghai, China). After detected the total protein concentrations by a bicinchoninic acid (BCA) protein assay (Beyotime, China), an equal amount of protein was resolved on 10% SDS-polyacrylamide gels (SDS-PAGE) and transferred to a polyvinylidene difluoride (PVDF) membrane for immunoblot analyses. The following primary antibodies were used to incubate proteins overnight at 4°C: goat anti-FSTL1, rabbit anti- iNOS, rabbit anti-TNF-α, rabbit anti-MMP-13, rabbit anti- COX-2 (all 1:2000; Abcam, USA); rabbit anti-p38, rabbit anti-p-p38, rabbit anti- JNK, rabbit anti-p-JNK, rabbit anti- ERK1/2, rabbit anti-p-ERK1/2, rabbit anti-p65, mouse anti- IκBα, rabbit anti-p-IκBα (all 1:1000; Cell Signaling Technology, Inc., MA, USA). The protein bands on the PVDF membranes were washed then horseradish peroxidase-conjugated secondary antibody (1:10000) was added and the bands were visualized using a FluorChem E chemiluminescent imaging system (Amersham Imager 600, General Electric Company, USA). ImageJ software (National Institutes of Health, USA) was used for densitometry analysis.

### Real-time RT-PCR

Total RNA was isolated from NP cells using the RNeasy Kit (Qiagen, Basel, Switzerland). Reverse transcription was performed using a real-time RT-PCR kit (Toyobo, Osaka, Japan) according to the manufacturer's instructions. Real-time PCR was performed with SYBR Green I dye which is used to monitor DNA synthesis. RT-PCR reactions were carried out on a Roche LightCycler (Roche, USA). Target gene expression was normalized to GAPDH. For each target gene, the experiment was repeated three times. Sequence-specific primers are listed in Table 2.

**Table 2:** Real-time PCR primers

Target gene	Forward primers, 5′–3′	Reverse primers, 5′–3′
**COX-2**	TTCTGA GATTGTGGGAAAATTGCT	AGATCATCTCTGGC GAGTATCTT
**IL-1β**	CTGTCCTGCGTGTTGAGGGA	TTGGGTAATTTTTGGGATCTACA
**IL-6**	TGGGCACAGAACTTATGTTG	TTGAGGTAAGCCTACACTTTCC
**iNOS**	ACGTCATAGTCTCTCTAAACCGTGC	GTGCTGACTGGAAATCTCAAGG
**MMP-13**	AAATTATGGAGGAGGAGATGCCCATT	TCCTTGGAGTGGTCAAGACCTAA
**TNF-α**	ATCTTCTCGAACCCCGAGTGA	GGAGCTGCCCCTCAGCTT
**GAPDH**	GGAGCGAGATCCCTCCAAAAT	GGCTGTTGTCATACTTCTCATGC

### Statistical analyses

Data are expressed as the mean ± SD. Statistical significance was determined by a one-way analysis of variance (ANOVA) with post hoc analysis. All tests were performed using SPSS 19.0 software (SPSS Inc., Chicago, IL, USA) and differences with P < 0.05 were considered to be statistically significant. The Spearman correlation coefficient ρ was used to analyze the correlation between VAS and serum FSTL1 levels.

## CONCLUSIONS

FSTL1 serum levels increased significantly in LDH patients. FSTL1 expression levels increased significantly in the degeneration of intervertebral discs in both humans and rats models. FSTL1 promoted disc inflammation by activation of JNK/MAPK, ERK1/2/MAPK, and NFκB signaling *in vitro*, suggesting that FSTL1 might be one potential novel agent for the treatment of inflammation in LDH.
